# Methyl 2-[(3*RS*,4*RS*)-3-phenyl-4-(phenyl­sulfon­yl)isoxazolidin-2-yl]acetate

**DOI:** 10.1107/S1600536814012161

**Published:** 2014-05-31

**Authors:** Zeynep Gültekin, Mehmet Civan, Wolfgang Frey, Tuncer Hökelek

**Affiliations:** a Department of Chemistry, Çankırı Karatekin University, TR-18100 Çankırı, Turkey; b Department of Physics, Hacettepe University, 06800 Beytepe, Ankara, Turkey; c Universität Stuttgart, Pfaffenwaldring 55, D-70569 Stuttgart, Germany

## Abstract

In the title compound, C_18_H_19_NO_5_S, the five-membered isoxazolidine ring is in a half-chair conformation, and the phenyl rings are oriented at a dihedral angle of 66.53 (3)°. In the crystal, C—H⋯O hydrogen bonds link the mol­ecules into a three-dimensional supra­molecular structure. A weak C—H⋯π inter­action is also observed between adjacent mol­ecules.

## Related literature   

For 1,3-dipolar cyclo­addition of nitro­nes with olefins leading to isoxazolidines, see: Gothelf & Jorgensen (1994 ▶); Gothelf *et al.* (1996 ▶); Cicchi *et al.* (2003 ▶). For the use of isoxazolidines in the syntheses of nucleosides, amino acids, peptides and β-lactams, see: Merino *et al.* (1998 ▶); Leggio *et al.* (1997 ▶); Langlois & Rakotondradany (2000 ▶); Hermkens *et al.* (1994 ▶); Tran *et al.* (2013 ▶). For the synthesis of (*Z*)-*N*-benzyl­idene-2-meth­oxy-2-oxoethanamine oxide, see: Diez-Martinez *et al.* (2010 ▶). For bond-length data, see: Allen *et al.* (1987 ▶). For ring puckering parameters, see: Cremer & Pople (1975 ▶).
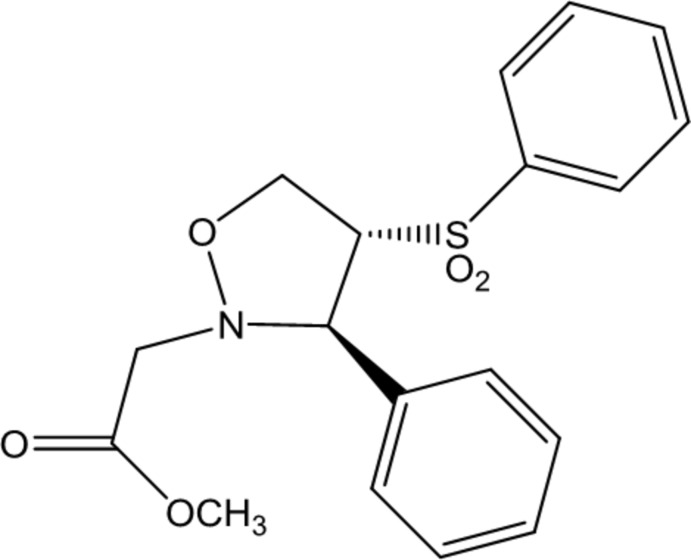


## Experimental   

### 

#### Crystal data  

C_18_H_19_NO_5_S
*M* *_r_* = 361.40
Monoclinic, 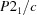

*a* = 8.2346 (2) Å
*b* = 15.1469 (5) Å
*c* = 13.7410 (4) Å
β = 103.362 (3)°
*V* = 1667.50 (8) Å^3^
*Z* = 4
Mo *K*α radiation
μ = 0.22 mm^−1^
*T* = 100 K
0.50 × 0.47 × 0.37 mm

#### Data collection  

Bruker Kappa APEXII DUO diffractometer
Absorption correction: multi-scan (Blessing, 1995 ▶) *T*_min_ = 0.896, *T*_max_ = 0.922
34342 measured reflections
5105 independent reflections
4839 reflections with *I* > 2σ(*I*)
*R*_int_ = 0.022

#### Refinement  

*R*[*F* ^2^ > 2σ(*F* ^2^)] = 0.031
*wR*(*F* ^2^) = 0.085
*S* = 1.03
5105 reflections
228 parameters
H-atom parameters constrained
Δρ_max_ = 0.44 e Å^−3^
Δρ_min_ = −0.32 e Å^−3^

### 

Data collection: *APEX2* (Bruker, 2008 ▶); cell refinement: *SAINT* (Bruker, 2008 ▶); data reduction: *SAINT*; program(s) used to solve structure: *SHELXS97* (Sheldrick, 2008 ▶); program(s) used to refine structure: *SHELXL97* (Sheldrick, 2008 ▶); molecular graphics: *ORTEP-3 for Windows* (Farrugia, 2012 ▶); software used to prepare material for publication: *WinGX* (Farrugia, 2012 ▶) and *PLATON* (Spek, 2009 ▶).

## Supplementary Material

Crystal structure: contains datablock(s) I, global. DOI: 10.1107/S1600536814012161/xu5792sup1.cif

Structure factors: contains datablock(s) I. DOI: 10.1107/S1600536814012161/xu5792Isup2.hkl

Supporting information file. DOI: 10.1107/S1600536814012161/xu5792Isup3.cml

CCDC reference: 1005309

Additional supporting information:  crystallographic information; 3D view; checkCIF report

## Figures and Tables

**Table 1:** Hydrogen-bond geometry (Å, °) *Cg*1 is the centroid of the C7–C12 ring.

*D*—H⋯*A*	*D*—H	H⋯*A*	*D*⋯*A*	*D*—H⋯*A*
C2—H2⋯O4^i^	1.00	2.32	3.2778 (11)	159
C14—H14⋯O2^ii^	0.95	2.48	3.3326 (11)	150
C15—H15⋯O3^iii^	0.95	2.60	3.4543 (12)	150
C18—H18⋯O5^iv^	0.95	2.50	3.4401 (12)	169
C6—H6*B*⋯*Cg*1^v^	0.98	2.74	3.6157 (12)	149

Symmetry codes: (i) 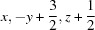; (ii) 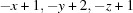; (iii) 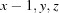; (iv) 
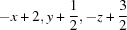
; (v) 
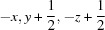
.

**Fig. 1.**
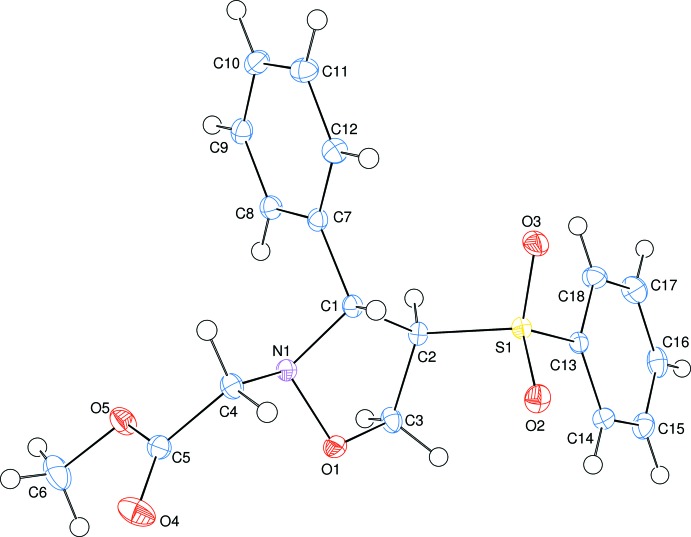
The molecular structure of the title compound with the atom-numbering scheme. Displacement ellipsoids are drawn at the 50% probability level.

**Fig. 2.**
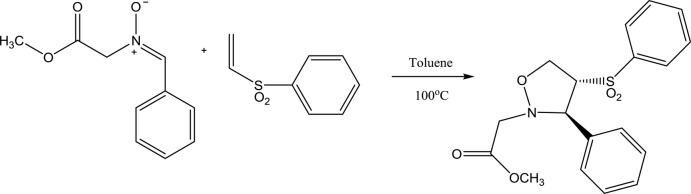
The reaction scheme.

## supplementary crystallographic information

### 1. Comment

1,3-dipolar cycloaddition of nitrones with olefines leads to isoxazolidines (Gothelf & Jorgensen, 1994; Gothelf *et al.*, 1996; Cicchi *et al.*, 2003). Isoxazolidines have been used for the syntheses of nucleosides (Merino *et al.*, 1998; Leggio *et al.*, 1997), amino acids (Langlois & Rakotondradany, 2000), peptides (Hermkens *et al.*, 1994) and β-lactams (Tran *et al.*, 2013). The title compound can be a useful intermediate for the preparation of 1,3-aminoalcohols in organic chemistry. The present study was undertaken to ascertain the crystal , structure of the title compound.

In the molecule of the title compound (Fig. 1) the bond lengths are within normal ranges (Allen *et al.*, 1987). The five-membered isoxazolidine ring [*C* (O1/N1/C1–C3)] is in half-chair conformation with puckering parameter (Cremer & Pople, 1975) of φ = -161.72 (6)°. The phenyl rings [*A* (C7–C12) and *B* (C13–C18)] are oriented at a dihedral angle of 66.53 (3)°. C1 and S1 atoms are -0.0301 (8) and -0.0326 (2) Å away from the corresponding planes of the phenyl rings *A* and *B*, respectively.

In the crystal structure, intermolecular C-H···O hydrogen bonds (Table 1) link the molecules into a three-dimensional structure, in which they may be effective in the stabilization of the structure. π···π contact between the phenyl rings, Cg1—Cg2^i^ [symmetry code: (i) - *x*, 1/2 + *y*, 1/2 - *z*, where Cg1 and Cg2 are the centroids of the rings *A* and *B*, respectively] may further stabilize the structure, with centroid-centroid distance of 3.9100 (5) Å. A weak C–H···π interaction (Table 1) has also been observed.

### 2. Experimental

The starting material, (Z)-N-benzylidene-2-methoxy-2-oxoethanamine oxide, was prepared by the literature method (Diez-Martinez *et al.*, 2010). For the synthesis of the title compound, (Z)-N-benzylidene-2-methoxy-2-oxo- ethanamine oxide (0.117 g, 0.605 mmol) was dissolved in toluene (2 ml), and then phenyl vinyl sulfone (0.102 g, 0.605 mmol) was added. The mixture was heated at 273 K for 5 h until the starting material was completely consumed as monitored by tlc. The resultant residue was directly purified by flash chromatography on silica using ethyl acetate as solvent. Crystallization of the product in ethyl acetate gave a colorless crystalline solid (yield: 92%), m.p.: 400-401 K.

### Crystal data

**Table:**

C_18_H_19_NO_5_S	*F*(000) = 760
*M**_r_* = 361.40	*D*_x_ = 1.440 Mg m^−^^3^
Monoclinic, *P*2_1_/*c*	Mo *K*α radiation, λ = 0.71073 Å
Hall symbol: -P 2ybc	Cell parameters from 4839 reflections
*a* = 8.2346 (2) Å	θ = 2.0–30.6°
*b* = 15.1469 (5) Å	µ = 0.22 mm^−^^1^
*c* = 13.7410 (4) Å	*T* = 100 K
β = 103.362 (3)°	Block, colourless
*V* = 1667.50 (8) Å^3^	0.50 × 0.47 × 0.37 mm
*Z* = 4	

### Data collection

**Table:**

Bruker Kappa APEXII DUO diffractometer	5105 independent reflections
Radiation source: fine-focus sealed tube	4839 reflections with *I* > 2σ(*I*)
Triumph monochromator	*R*_int_ = 0.022
ω + Phi Scans scans	θ_max_ = 30.6°, θ_min_ = 2.0°
Absorption correction: multi-scan (Blessing, 1995)	*h* = −11→11
*T*_min_ = 0.896, *T*_max_ = 0.922	*k* = −21→21
34342 measured reflections	*l* = −19→19

### Refinement

**Table:**

Refinement on *F*^2^	Secondary atom site location: difference Fourier map
Least-squares matrix: full	Hydrogen site location: inferred from neighbouring sites
*R*[*F*^2^ > 2σ(*F*^2^)] = 0.031	H-atom parameters constrained
*wR*(*F*^2^) = 0.085	*w* = 1/[σ^2^(*F*_o_^2^) + (0.0474*P*)^2^ + 0.6732*P*] where *P* = (*F*_o_^2^ + 2*F*_c_^2^)/3
*S* = 1.03	(Δ/σ)_max_ = 0.003
5105 reflections	Δρ_max_ = 0.44 e Å^−^^3^
228 parameters	Δρ_min_ = −0.32 e Å^−^^3^
0 restraints	Extinction correction: *SHELXL97* (Sheldrick, 2008), Fc^*^=kFc[1+0.001xFc^2^λ^3^/sin(2θ)]^-1/4^
Primary atom site location: structure-invariant direct methods	Extinction coefficient: 0.0182 (13)

### Special details

**Table:**

Geometry. All esds (except the esd in the dihedral angle between two l.s. planes) are estimated using the full covariance matrix. The cell esds are taken into account individually in the estimation of esds in distances, angles and torsion angles; correlations between esds in cell parameters are only used when they are defined by crystal symmetry. An approximate (isotropic) treatment of cell esds is used for estimating esds involving l.s. planes.
Refinement. Refinement of F^2^ against ALL reflections. The weighted R-factor wR and goodness of fit S are based on F^2^, conventional R-factors R are based on F, with F set to zero for negative F^2^. The threshold expression of F^2^ > 2sigma(F^2^) is used only for calculating R-factors(gt) etc. and is not relevant to the choice of reflections for refinement. R-factors based on F^2^ are statistically about twice as large as those based on F, and R- factors based on ALL data will be even larger.

### Fractional atomic coordinates and isotropic or equivalent isotropic displacement parameters (Å^2^)

**Table:**

	*x*	*y*	*z*	*U*_iso_*/*U*_eq_	
S1	0.79940 (2)	1.032574 (13)	0.635211 (14)	0.01200 (6)	
O1	0.70058 (8)	0.81466 (4)	0.54622 (5)	0.01522 (13)	
O2	0.72422 (9)	1.03940 (4)	0.52956 (5)	0.01772 (13)	
O3	0.96262 (8)	1.07049 (5)	0.67213 (5)	0.01888 (14)	
O4	0.80821 (10)	0.63499 (6)	0.40190 (6)	0.02603 (16)	
O5	0.86652 (9)	0.60662 (5)	0.56648 (5)	0.02053 (14)	
N1	0.86900 (9)	0.78645 (5)	0.59648 (5)	0.01279 (13)	
C1	0.95186 (10)	0.87109 (5)	0.63373 (6)	0.01223 (14)	
H1	0.9807	0.9049	0.5775	0.015*	
C2	0.81075 (10)	0.91865 (5)	0.67116 (6)	0.01263 (14)	
H2	0.8324	0.9140	0.7456	0.015*	
C3	0.65340 (11)	0.86529 (6)	0.62291 (7)	0.01560 (16)	
H3A	0.6202	0.8262	0.6727	0.019*	
H3B	0.5592	0.9051	0.5942	0.019*	
C4	0.93803 (11)	0.74795 (6)	0.51777 (6)	0.01446 (15)	
H4A	1.0605	0.7420	0.5417	0.017*	
H4B	0.9157	0.7877	0.4590	0.017*	
C5	0.86226 (10)	0.65792 (6)	0.48709 (7)	0.01502 (16)	
C6	0.79515 (15)	0.51924 (7)	0.54660 (9)	0.0275 (2)	
H6A	0.6763	0.5243	0.5143	0.041*	
H6B	0.8082	0.4869	0.6097	0.041*	
H6C	0.8527	0.4874	0.5023	0.041*	
C7	1.10686 (10)	0.85329 (5)	0.71446 (6)	0.01261 (15)	
C8	1.10257 (11)	0.79292 (6)	0.79082 (6)	0.01487 (15)	
H8	1.0023	0.7622	0.7915	0.018*	
C9	1.24526 (11)	0.77794 (6)	0.86569 (6)	0.01609 (16)	
H9	1.2423	0.7366	0.9172	0.019*	
C10	1.39254 (11)	0.82316 (6)	0.86564 (7)	0.01755 (17)	
H10	1.4898	0.8127	0.9169	0.021*	
C11	1.39663 (11)	0.88376 (6)	0.79017 (7)	0.01806 (17)	
H11	1.4963	0.9154	0.7905	0.022*	
C12	1.25445 (11)	0.89810 (6)	0.71397 (7)	0.01584 (16)	
H12	1.2583	0.9385	0.6617	0.019*	
C13	0.66058 (10)	1.07755 (5)	0.70249 (6)	0.01229 (14)	
C14	0.49136 (11)	1.08311 (6)	0.65773 (7)	0.01553 (16)	
H14	0.4499	1.0639	0.5908	0.019*	
C15	0.38351 (11)	1.11734 (6)	0.71286 (8)	0.01927 (17)	
H15	0.2674	1.1215	0.6835	0.023*	
C16	0.44528 (12)	1.14540 (6)	0.81051 (8)	0.01993 (18)	
H16	0.3711	1.1683	0.8480	0.024*	
C17	0.61525 (13)	1.14025 (7)	0.85386 (7)	0.02118 (18)	
H17	0.6568	1.1602	0.9205	0.025*	
C18	0.72483 (11)	1.10607 (6)	0.80006 (7)	0.01731 (16)	
H18	0.8410	1.1023	0.8293	0.021*	

### Atomic displacement parameters (Å^2^)

**Table:**

	*U*^11^	*U*^22^	*U*^33^	*U*^12^	*U*^13^	*U*^23^
S1	0.01266 (10)	0.01083 (10)	0.01277 (10)	0.00014 (6)	0.00347 (7)	−0.00027 (6)
O1	0.0121 (3)	0.0164 (3)	0.0166 (3)	0.0018 (2)	0.0022 (2)	−0.0024 (2)
O2	0.0230 (3)	0.0180 (3)	0.0123 (3)	0.0022 (2)	0.0046 (2)	0.0020 (2)
O3	0.0131 (3)	0.0166 (3)	0.0276 (3)	−0.0022 (2)	0.0059 (2)	−0.0041 (2)
O4	0.0288 (4)	0.0321 (4)	0.0171 (3)	−0.0077 (3)	0.0053 (3)	−0.0090 (3)
O5	0.0263 (3)	0.0146 (3)	0.0199 (3)	−0.0044 (2)	0.0036 (3)	−0.0014 (2)
N1	0.0122 (3)	0.0127 (3)	0.0134 (3)	0.0013 (2)	0.0028 (2)	−0.0020 (2)
C1	0.0137 (3)	0.0113 (3)	0.0120 (3)	0.0005 (3)	0.0036 (3)	−0.0004 (3)
C2	0.0143 (3)	0.0112 (3)	0.0130 (3)	0.0014 (3)	0.0044 (3)	0.0007 (3)
C3	0.0151 (4)	0.0130 (3)	0.0204 (4)	−0.0004 (3)	0.0076 (3)	−0.0018 (3)
C4	0.0162 (4)	0.0142 (4)	0.0139 (3)	0.0003 (3)	0.0054 (3)	−0.0020 (3)
C5	0.0122 (3)	0.0170 (4)	0.0165 (4)	0.0008 (3)	0.0047 (3)	−0.0037 (3)
C6	0.0315 (5)	0.0157 (4)	0.0376 (6)	−0.0070 (4)	0.0126 (4)	−0.0047 (4)
C7	0.0139 (3)	0.0118 (3)	0.0123 (3)	0.0014 (3)	0.0034 (3)	−0.0009 (3)
C8	0.0161 (4)	0.0140 (4)	0.0146 (3)	0.0000 (3)	0.0039 (3)	0.0004 (3)
C9	0.0193 (4)	0.0147 (4)	0.0142 (4)	0.0028 (3)	0.0037 (3)	0.0010 (3)
C10	0.0155 (4)	0.0203 (4)	0.0160 (4)	0.0038 (3)	0.0020 (3)	−0.0015 (3)
C11	0.0139 (4)	0.0218 (4)	0.0188 (4)	−0.0007 (3)	0.0045 (3)	−0.0013 (3)
C12	0.0154 (4)	0.0168 (4)	0.0161 (4)	−0.0006 (3)	0.0050 (3)	0.0005 (3)
C13	0.0129 (3)	0.0105 (3)	0.0138 (3)	0.0004 (3)	0.0036 (3)	−0.0004 (3)
C14	0.0137 (3)	0.0147 (4)	0.0173 (4)	0.0003 (3)	0.0018 (3)	0.0003 (3)
C15	0.0146 (4)	0.0162 (4)	0.0281 (5)	0.0016 (3)	0.0071 (3)	0.0018 (3)
C16	0.0234 (4)	0.0137 (4)	0.0268 (5)	0.0016 (3)	0.0141 (4)	0.0000 (3)
C17	0.0259 (4)	0.0212 (4)	0.0178 (4)	0.0000 (3)	0.0078 (3)	−0.0051 (3)
C18	0.0165 (4)	0.0192 (4)	0.0155 (4)	0.0004 (3)	0.0022 (3)	−0.0036 (3)

### Geometric parameters (Å, º)

**Table:**

S1—O2	1.4444 (7)	C6—H6C	0.9800
S1—O3	1.4417 (7)	C7—C12	1.3933 (12)
S1—C13	1.7646 (8)	C7—C8	1.3985 (12)
S1—C2	1.7914 (8)	C8—C9	1.3898 (12)
O1—N1	1.4630 (9)	C8—H8	0.9500
O1—C3	1.4278 (10)	C9—C10	1.3931 (13)
O4—C5	1.2034 (11)	C9—H9	0.9500
O5—C5	1.3333 (11)	C10—C11	1.3911 (13)
O5—C6	1.4483 (12)	C10—H10	0.9500
N1—C1	1.4865 (11)	C11—C12	1.3956 (12)
N1—C4	1.4551 (10)	C11—H11	0.9500
C1—C7	1.5092 (11)	C12—H12	0.9500
C1—C2	1.5524 (11)	C13—C14	1.3896 (11)
C1—H1	1.0000	C13—C18	1.3914 (12)
C2—C3	1.5410 (12)	C14—C15	1.3937 (13)
C2—H2	1.0000	C14—H14	0.9500
C3—H3A	0.9900	C15—C16	1.3871 (14)
C3—H3B	0.9900	C15—H15	0.9500
C4—C5	1.5182 (12)	C16—C17	1.3909 (14)
C4—H4A	0.9900	C16—H16	0.9500
C4—H4B	0.9900	C17—C18	1.3918 (13)
C6—H6A	0.9800	C17—H17	0.9500
C6—H6B	0.9800	C18—H18	0.9500
			
O2—S1—C2	109.21 (4)	O5—C6—H6C	109.5
O2—S1—C13	108.66 (4)	H6A—C6—H6B	109.5
O3—S1—O2	118.25 (4)	H6A—C6—H6C	109.5
O3—S1—C2	107.56 (4)	H6B—C6—H6C	109.5
O3—S1—C13	109.03 (4)	C8—C7—C1	120.30 (7)
C13—S1—C2	103.07 (4)	C12—C7—C1	119.99 (7)
C3—O1—N1	101.44 (6)	C12—C7—C8	119.70 (8)
C5—O5—C6	116.45 (8)	C7—C8—H8	120.0
O1—N1—C1	102.73 (6)	C9—C8—C7	119.90 (8)
C4—N1—O1	104.87 (6)	C9—C8—H8	120.0
C4—N1—C1	112.00 (7)	C8—C9—C10	120.43 (8)
N1—C1—C2	101.25 (6)	C8—C9—H9	119.8
N1—C1—C7	109.99 (7)	C10—C9—H9	119.8
N1—C1—H1	110.3	C9—C10—H10	120.1
C2—C1—H1	110.3	C11—C10—C9	119.73 (8)
C7—C1—C2	114.21 (7)	C11—C10—H10	120.1
C7—C1—H1	110.3	C10—C11—C12	120.09 (8)
S1—C2—H2	109.6	C10—C11—H11	120.0
C1—C2—S1	110.55 (5)	C12—C11—H11	120.0
C1—C2—H2	109.6	C7—C12—C11	120.14 (8)
C3—C2—S1	113.67 (6)	C7—C12—H12	119.9
C3—C2—C1	103.50 (6)	C11—C12—H12	119.9
C3—C2—H2	109.6	C14—C13—S1	119.75 (6)
O1—C3—C2	104.72 (6)	C14—C13—C18	121.79 (8)
O1—C3—H3A	110.8	C18—C13—S1	118.46 (6)
O1—C3—H3B	110.8	C13—C14—C15	118.80 (8)
C2—C3—H3A	110.8	C13—C14—H14	120.6
C2—C3—H3B	110.8	C15—C14—H14	120.6
H3A—C3—H3B	108.9	C14—C15—H15	119.9
N1—C4—C5	111.12 (7)	C16—C15—C14	120.16 (8)
N1—C4—H4A	109.4	C16—C15—H15	119.9
N1—C4—H4B	109.4	C15—C16—C17	120.32 (8)
C5—C4—H4A	109.4	C15—C16—H16	119.8
C5—C4—H4B	109.4	C17—C16—H16	119.8
H4A—C4—H4B	108.0	C16—C17—C18	120.34 (9)
O4—C5—O5	124.17 (9)	C16—C17—H17	119.8
O4—C5—C4	124.42 (9)	C18—C17—H17	119.8
O5—C5—C4	111.40 (7)	C13—C18—C17	118.58 (8)
O5—C6—H6A	109.5	C13—C18—H18	120.7
O5—C6—H6B	109.5	C17—C18—H18	120.7
			
O2—S1—C2—C1	74.55 (6)	C7—C1—C2—C3	−133.54 (7)
O2—S1—C2—C3	−41.32 (7)	N1—C1—C7—C8	−45.64 (10)
O3—S1—C2—C1	−54.94 (6)	N1—C1—C7—C12	135.35 (8)
O3—S1—C2—C3	−170.82 (6)	C2—C1—C7—C8	67.39 (10)
C13—S1—C2—C1	−170.06 (6)	C2—C1—C7—C12	−111.62 (9)
C13—S1—C2—C3	74.06 (6)	S1—C2—C3—O1	104.22 (7)
O2—S1—C13—C14	22.31 (8)	C1—C2—C3—O1	−15.74 (8)
O2—S1—C13—C18	−158.13 (7)	N1—C4—C5—O4	132.00 (9)
O3—S1—C13—C14	152.47 (7)	N1—C4—C5—O5	−49.40 (9)
O3—S1—C13—C18	−27.97 (8)	C1—C7—C8—C9	−179.03 (8)
C2—S1—C13—C14	−93.47 (7)	C12—C7—C8—C9	−0.02 (13)
C2—S1—C13—C18	86.09 (7)	C1—C7—C12—C11	178.06 (8)
C3—O1—N1—C1	−52.69 (7)	C8—C7—C12—C11	−0.95 (13)
C3—O1—N1—C4	−169.90 (6)	C7—C8—C9—C10	0.46 (13)
N1—O1—C3—C2	41.37 (7)	C8—C9—C10—C11	0.06 (13)
C6—O5—C5—O4	−2.30 (13)	C9—C10—C11—C12	−1.03 (14)
C6—O5—C5—C4	179.09 (8)	C10—C11—C12—C7	1.48 (14)
O1—N1—C1—C2	40.94 (7)	S1—C13—C14—C15	178.83 (7)
O1—N1—C1—C7	162.10 (6)	C18—C13—C14—C15	−0.72 (13)
C4—N1—C1—C2	152.97 (7)	S1—C13—C18—C17	−178.97 (7)
C4—N1—C1—C7	−85.88 (8)	C14—C13—C18—C17	0.59 (14)
O1—N1—C4—C5	−74.22 (8)	C13—C14—C15—C16	0.17 (13)
C1—N1—C4—C5	175.10 (7)	C14—C15—C16—C17	0.50 (14)
N1—C1—C2—S1	−137.47 (5)	C15—C16—C17—C18	−0.64 (15)
N1—C1—C2—C3	−15.40 (8)	C16—C17—C18—C13	0.10 (14)
C7—C1—C2—S1	104.39 (7)		

### Hydrogen-bond geometry (Å, º)

Cg1 is the centroid of the C7–C12 ring.

**Table:**

*D*—H···*A*	*D*—H	H···*A*	*D*···*A*	*D*—H···*A*
C2—H2···O4^i^	1.00	2.32	3.2778 (11)	159
C14—H14···O2^ii^	0.95	2.48	3.3326 (11)	150
C15—H15···O3^iii^	0.95	2.60	3.4543 (12)	150
C18—H18···O5^iv^	0.95	2.50	3.4401 (12)	169
C6—H6*B*···*Cg*1^v^	0.98	2.74	3.6157 (12)	149

Symmetry codes: (i) *x*, −*y*+3/2, *z*+1/2; (ii) −*x*+1, −*y*+2, −*z*+1; (iii) *x*−1, *y*, *z*; (iv) −*x*+2, *y*+1/2, −*z*+3/2; (v) −*x*, *y*+1/2, −*z*+1/2.
